# Microtubules in Pancreatic β Cells: Convoluted Roadways Toward Precision

**DOI:** 10.3389/fcell.2022.915206

**Published:** 2022-07-08

**Authors:** Kai M. Bracey, Guoqiang Gu, Irina Kaverina

**Affiliations:** Department of Cell and Developmental Biology and Vanderbilt Program in Developmental Biology, Vanderbilt University, Nashville, TN, United States

**Keywords:** microtubule, insulin, pancreatic beta cell, endocrine cell, kinesin, dynein, glucose-stimulated insulin secretion

## Abstract

Pancreatic islet β cells regulate glucose homeostasis *via* glucose-stimulated insulin secretion (GSIS). Cytoskeletal polymers microtubules (MTs) serve as tracks for the transport and positioning of secretory insulin granules. MT network in β cells has unique morphology with several distinct features, which support granule biogenesis (*via* Golgi-derived MT array), net non-directional transport (*via* interlocked MT mesh), and control availability of granules at secretion sites (*via* submembrane MT bundle). The submembrane MT array, which is parallel to the plasma membrane and serves to withdraw excessive granules from the secretion hot spots, is destabilized and fragmented downstream of high glucose stimulation, allowing for regulated secretion. The origin of such an unusual MT network, the features that define its functionality, and metabolic pathways that regulate it are still to a large extent elusive and are a matter of active investigation and debate. Besides the MT network itself, it is important to consider the interplay of molecular motors that drive and fine-tune insulin granule transport. Importantly, activity of kinesin-1, which is the major MT-dependent motor in β cells, transports insulin granules, and has a capacity to remodel MT network, is also regulated by glucose. We discuss yet unknown potential avenues toward understanding how MT network and motor proteins provide control for secretion in coordination with other GSIS-regulating mechanisms.

## Introduction

Pancreatic islet β cells regulate glucose homeostasis in vertebrates *via* glucose-stimulated insulin secretion (GSIS). This function is of critical importance to human health, because excessive GSIS causes hyperinsulinemic hypoglycemia that damages the brain and other tissues (Nessa et al., 2016), while insufficient secretion causes diabetes (Hudish et al., 2019; Alejandro et al., 2015). To avoid these pathological effects, the correct number of secretion-competent insulin granules (IGs) has to be prepared prior to a glucose stimulus. Moreover, these IGs must be positioned to the exocytosis sites at the plasma membrane at the right moment (Gao et al., 2007; Kasai et al., 2008; Verhage and Sorensen, 2008; Wang and Thurmond, 2009; Zhao et al., 2010; Gandasi et al., 2018). Several studies over the years have established that microtubules (MTs), which serve as tracks for IG transport, precisely regulate IG positioning over time (Heaslip et al., 2014; Hoboth et al., 2015; Zhu et al., 2015; Bracey et al., 2020; Ho et al., 2020; Trogden et al., 2021).

Our surprising findings are that the β-cell MTs serve two functions. In the long-term, they promote endocrine function *via* facilitating IG biosynthesis, which relies on the growth of new MTs (Trogden et al., 2019). In the short-term, they acutely attenuate GSIS by restricting the number of readily-releasable IGs at the secretion sites, depending on MT stabilization (Zhu et al., 2015; Bracey et al., 2020; Ho et al., 2020; Trogden et al., 2021). In this perspective, we will provide our current views on how MT networks in β cells are designed at the cellular and sub-cellular scales to precisely tune IG-transport and GSIS. Our current model is that β-cell MTs are built, modulated, and utilized by such intracellular factors as MT-associated proteins (MAPs) and molecular motors to regulate IG transport and secretion.

## Challenge of Insulin Granule Transport for Correct Secretion Levels

The task of correctly positioning secretory vesicles for acute stimulated secretion is a complicated process. On one hand, IGs are produced from the trans-Golgi network in the inner cytoplasm (Hou et al., 2009). They must then be transported to underneath the plasma membrane for regulated release (Gao et al., 2007; Kasai et al., 2008; Verhage and Sorensen, 2008; Wang and Thurmond, 2009; Zhao et al., 2010; Gandasi et al., 2018). Since most of the IG transport in a β cell is MT-dependent (Varadi et al., 2002; Tabei et al., 2013; Heaslip et al., 2014; Zhu et al., 2015), overall IG distribution in the cytoplasm including the cell periphery is a result of such active transport.

On the other hand, insulin secretion occurs within minutes after high glucose treatment, while it takes hours for newly synthesized IGs to mature and reach the cell membrane (Hou et al., 2009). To provide a timely response, a large number of pre-processed secretion-competent IGs is awaiting the signal. In a resting β cell, several thousands of IGs fill the entire cytoplasm, and only ∼4% of those are secreted at a given stimulus (Dean, 1973; Olofsson et al., 2002; Rorsman and Renstrom, 2003; Fu et al., 2013). This scenario dramatically differs from constitutive secretion when secretory vesicles are constantly produced and readily transported for immediate secretion.

Not surprisingly, numerous secretion-restricting mechanisms have evolved to prevent over-secretion *via* the occasional release of pre-stored IGs (Chatterjee Bhowmick et al., 2021). These mechanisms act in concert as a combination of locks on a door or filters in a pipe. Broadly defined, these “filters” include any cellular tool preventing uncontrollable secretion. For example, restricting calcium levels in the cytoplasm can be considered a filter, because calcium influx is needed for priming of docked IGs for secretion (Idevall-Hagren and Tengholm, 2020; Omar-Hmeadi and Idevall-Hagren, 2021). Actin cytoskeleton provides another set of filters: actin remodeling and activity of myosins are thought to remove steric hindrance between IGs and the plasma membrane, drive short-distance IG transport to the secretion site, and provide mechanical force for the exocytic event itself (Varadi et al., 2005; Kalwat and Thurmond, 2013; Arous and Halban, 2015; Veluthakal and Thurmond, 2021). When we discuss MT-dependent positioning and transport of IGs in the cytoplasm, we must take the over-crowding of β-cell cytoplasm into account and consider that removal of IG from the secretion sites can act as one of those “filters”. Our findings over the last few years support this model (Zhu et al., 2015; Bracey et al., 2020; Ho et al., 2020; Trogden et al., 2021). All the “filters” that prevent secretion at a steady-state (basal) conditions must be adjusted upon each stimulus to allow a proper number of IGs to be secreted (Idevall-Hagren and Tengholm, 2020; Omar-Hmeadi and Idevall-Hagren, 2021). Being one of the filters, the MT network and transport must be modified downstream of glucose in a precise and reversable manner. Indeed, emerging evidence indicates that both MT network itself and MT-dependent motor activity are regulated by glucose signaling in β cells (Donelan et al., 2002; McDonald et al., 2009; Heaslip et al., 2014; Zhu et al., 2015; Trogden et al., 2019; Ho et al., 2020; Trogden et al., 2021).

## Organization and Origin of Microtubule Network in β Cells

**FIGURE 1:**
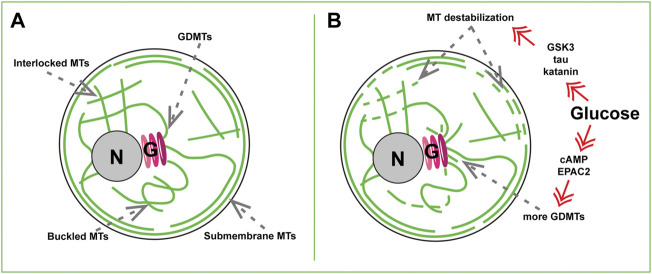
MT sub-populations in a beta cell. **(A)** Steady-state basal glucose conditions. Newly nucleated GDMTs and the Golgi (G), Inner MT mesh formed by interlocked and buckled MTs, and submembrane MT array are shown. **(B)** High-glucose triggered MT remodeling *via* two signaling axes. Top: Kinase- (e.g. GSK3), tau phosphorylation-, and katanin severing-dependent MT destabilization. Bottom: cAMP and EPAC2- dependent enhancement of GDMT nucleation. Nucleus, N.

Early work has assumed that MT networks in β cells resemble radial MT organization in other cells (Byers et al., 1980), and serve for directional delivery of IGs to the cell periphery (Lacy et al., 1972). Such a view emerged, in part, due to technical inability, at that time, to distinguish which MT networks belonged to functional β cells vs. other cell types in pancreatic primary cell cultures (Boyd et al., 1982). This view has been challenged by the demonstration of a complex MT network in Min6 cells (Varadi et al., 2003), followed by a series of data in primary functional β cells uncovering a dense non-radial interlocking mesh of MTs in mouse and human islet β cells (Figure 1A) (Zhu et al., 2015; Trogden et al., 2019; Bracey et al., 2020; Ho et al., 2020; Muller et al., 2021). Identification of the sites of MT origin (nucleation) has shown that the vast majority of β-cell MTs nucleates at the Golgi complex membrane (Figure 1A) (Zhu et al., 2015; Trogden et al., 2019). Such MTs, in contrast to those nucleated at the conventional MT-organizing centers (MTOCs), the centrosomes, are called Golgi-derived MTs, or GDMTs (Zhu and Kaverina, 2013; Sanders and Kaverina, 2015). More recently, a thorough analysis of three-dimensional confocal (Bracey et al., 2020) and electron microscopy (Muller et al., 2021) data has shown that in addition to inner meshwork, the islet β-cell MT network features a prominent array of peripheral MTs parallel to the plasma membrane (Figure 1A). We found that this sub-membrane MT bundle is locally stabilized by a MT-associated protein (MAP) tau (Ho et al., 2020).

What could possibly produce a MT network so drastically different from the classic radial MT array? Switching MT nucleation to the Golgi can, in part, explain the non-radial MT pattern in the β-cell interior: because the Golgi in β cells is relatively large, GDMT minus ends are intrinsically distributed over a significant volume, contributing to the network complexity. In addition, irregular MT organization might result from their extended lifetimes: β-cell MTs are extraordinarily stable with a half-life of hours at basal glucose compared with minutes to tens of minutes in other cell types (Zhu et al., 2015; Ho et al., 2020). This increases their flexibility (Portran et al., 2017) and the probability of bending and buckling by intracellular forces over time (Odde et al., 1999; Straube et al., 2006; Bicek et al., 2009).

The origin of the sub-membrane MT bundle is, so far, a matter of speculation. GDMTs might extend from the Golgi to the cell periphery and bend to elongate along the plasma membrane (Figure 1A), however such long GDMTs have not yet been detected in β cells (Muller et al., 2021). Nevertheless, MTs do not nucleate anew at β-cell locations other than the Golgi, and, to a much lesser extent, the centrosome (Trogden et al., 2019), meaning that GDMTs must serve as precursors of most components of the MT network in a long run. In principle, MT polymer mass can increase without new nucleation, *via* using small fragments of existing MTs as seeds (Roll-Mecak and Vale, 2006). Interestingly, FIB-SEM studies found multiple small MT fragments at the β-cell periphery (Muller et al., 2021), which might serve as such seeds. These fragments were suggested to potentially arise from MT severing by katanin-family proteins directly in the sub-membrane area (Muller et al., 2021). Alternatively, these fragments could be short MTs nucleated elsewhere (most likely, at the Golgi) and transported to the cell periphery by a motor-dependent MT sliding, as described in other cell types (Jolly and Gelfand, 2010).

On an additional note, it is important to keep in mind that everything said above assumes that every β cell has a similar MT organization. This, however, is not the case. β cells are known to be extremely heterogeneous in their granularity, Ca^2+^ response, metabolic activity, GSIS level, and gene expression (Avrahami et al., 2017; Nasteska and Hodson, 2018; Miranda et al., 2021; Benninger and Kravets, 2022). Not an exception, MTs also vary significantly from one β cell to another, as obvious from the dramatic differences in the amount of long-lived MTs, detected by the detyrosinated tubulin immunostaining (Trogden et al., 2021). This means that certain β cells have stable, unchanging MT networks, while others have more labile, dynamic networks. Thus, some important subtypes of β-cell MT networks might potentially differ from the generalized picture described here. Moreover, specific local MT features within individual cells might have yet escaped averaged analyses (Bracey et al., 2020) or studies unavoidably restricted to a small sample number [e.g., FIB-SEM, (Muller et al., 2021)], and may be functionally very important. Thus, the heterogeneity of MTs within the β-cell population and their fine functional features remain an intriguing area of research.

## β-Cell Microtubule Network Regulation by Glucose

The critical features of β cells are to be able to respond to postprandial glucose stimulus properly and rapidly and to be able to revert to a steady-state condition after the glucose levels have been reduced. Like other important β-cellular systems, the MT network readily reacts to stimulation. Combined evidence (Heaslip et al., 2014; Zhu et al., 2015; Trogden et al., 2019; Ho et al., 2020; Muller et al., 2021) indicates that being very stable at basal, low-glucose, conditions, MTs undergo a significant turnover in high glucose: both destabilization/depolymerization of pre-existing MTs and simultaneous polymerization of new MTs (Figure 1B).

Live imaging assays indicate that MT depolymerization is triggered already 5 min after the high glucose application (Ho et al., 2020). This response relies on hyper-phosphorylation of MT stabilizer tau *via* glucose-responsive kinases GSK3, PKA, PKC, and CDK5 (Ho et al., 2020), which promotes tau detachment from MTs (Lindwall and Cole, 1984). While dynamics of the whole MT network is facilitated upon tau removal, this effect is especially manifested at the submembrane MTs (Figure 1B), which initially contain a higher concentration of tau (Ho et al., 2020). This glucose-dependent MT destabilization coincides with a substantial fragmentation of MTs into small “seeds” (Muller et al., 2021), which may be potentially used to rebuild the submembrane bundle after glucose is cleared from the extracellular media. It is tempting to suggest that tau hyper-phosphorylation and detachment from MTs is a priming step for submembrane MT severing by katanin [as suggested in (Muller et al., 2021)], considering that tau is known to protect MTs from such severing in neurons or *in vitro* (Barbier et al., 2019).

In parallel with destabilization, glucose stimulation also leads to an increase in MT polymerization. This includes facilitated MT nucleation at the Golgi [Figure 1B, (Trogden et al., 2019)] and faster polymerization at peripheral MT plus ends (Heaslip et al., 2014). Such responses likely compensate for MT loss, so that the whole MT polymer mass is affected by glucose only to a slight (Zhu et al., 2015) or non-detectable level (Muller et al., 2021). At the same time, the amount of detyrosinated tubulin (Glu-tubulin) within MTs, a post-translational modification used as a readout for MT lifetime (Gundersen et al., 1987; Khawaja et al., 1988), is significantly decreased (Zhu et al., 2015; Ho et al., 2020; Trogden et al., 2021), which is a direct indication of high MT polymer turnover.

Intriguingly, signaling pathways downstream of glucose that trigger the processes of MT depolymerization versus repolymerization in β cells are distinct from one another. While MT destabilization is ATP-production and kinase-dependent (Ho et al., 2020), MT nucleation at the β-cell Golgi requires cAMP and cAMP effector EPAC2 (Trogden et al., 2019), another metabolic signaling axis involved in GSIS (Renstrom et al., 1997; Ramos et al., 2008). This suggests that the amount of MT polymer can be fine-tuned by relative inputs of those two pathways. In addition, MT subsets predominantly affected by those pathways are also distinct, meaning that while the MT polymer mass is mostly sustained, the balance between different MT subsets is likely tilted at the time of secretion stimulation. We will next discuss how changing specific MT subsets and their relative representation within a cell affects β-cell function and fitness.

## Functions of Distinct Subpopulations of β-Cell Microtubule Network

Complete depolymerization of MTs by nocodazole leads to enhanced GSIS (Zhu et al., 2015; Trogden et al., 2021), laying a base for our model that MTs serve as one of the “filters” for regulated, dosed secretion levels. What are the functions of distinct MT subsets and how do they affect secretion?

**FIGURE 2:**
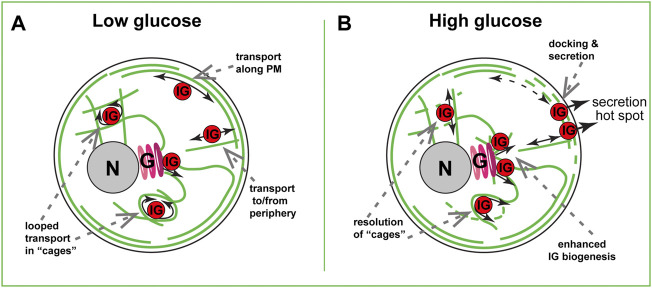
MT-dependent IG transport in a beta cell. **(A)** Steady-state basal glucose conditions. Low number of nascent IGs are formed at the Golgi (G) in a GDMTs-dependent manner. In the inner cytoplasm, many IGs are trapped in MT cages and undergo looped transport. At the cell periphery, sub-membrane and randomly oriented MTs serve for bi-directional IG transport. **(B)** High-glucose triggered IG transport. Partial destabilization of inner meshwork allows for the resolution of “cages” and rare directional transport events. Local destabilization of submembrane MTs allows for interruption of IG withdrawal from the hot spots, leading to docking and secretion. Facilitated GDMT formation supports new IG biogenesis to replenish the IG population. Golgi, (G). Nucleus, N. Plasma membrane, PM.

The net movement of insulin granules is non-directional and has characteristics of sub-diffusion, or random walk (Tabei et al., 2013; Zhu et al., 2015). This is, however, not true diffusion: the β-cell cytoplasm is too crowded for IG to move unless they are forcefully transported by molecular motors. Non-directional transport likely arises from a convoluted configuration of MT tracks in the β-cell interior (Varadi et al., 2003; Zhu et al., 2015), where IGs frequently switch tracks and/or follow buckled MT loops (Figure 2A). In addition, IGs likely often get restrained by the dense MT meshwork and other components of the crowded cytoplasm.

In the absence of other factors, sub-diffusive transport should move IGs down the concentration gradient: from the areas of high IG abundance to the areas of low IG abundance. Under conditions of IG depletion at the periphery after an extreme secretion wave (degranulation), this would deliver IGs from the cell interior to the cell periphery [positive MT regulation of secretion, as proposed in (Lacy et al., 1972; Boyd et al., 1982; Rorsman and Renstrom, 2003)]. Net IG transport in a healthy β cell does not enrich IG concentration at the cell periphery (Zhu et al., 2015), probably due to an ample IG abundance at that location (the lack of IG gradient). However, instances of direct IG movement along MTs (Figure 2A) have been reported (Varadi et al., 2002; Heaslip et al., 2014; Hoboth et al., 2015). Such rare events deliver recently produced (young) insulin granules toward the periphery for secretion regardless of the gradient and have been proposed to serve as a positive MT regulation of secretion in functional β cells (Hoboth et al., 2015).

Instances of direct IG movement along MTs were also reported when observing IG transport by TIRF microscopy, which by design visualizes only peripheral, submembrane IGs (Varadi et al., 2003; Zhu et al., 2015). Such movements are consistent with utilizing peripheral MTs parallel to the membrane (Figure 2A, (Bracey et al., 2020). Those submembrane MTs get destabilized and fragmented upon glucose stimulation (Ho et al., 2020; Muller et al., 2021), prompting a hypothesis that this MT array must be interrupted for secretion to occur (Figure 2B). Indeed, preventing MT destabilization by taxol treatment inhibits GSIS (Howell et al., 1982; Zhu et al., 2015). Our computational model predicts that submembrane MTs, as long as they are connected with the inner MT network and serve as tracks for bi-directional IG transport, will promote the removal of IGs from the periphery, acting like a “sponge” (Bracey et al., 2020).

Interestingly, both computational (Bracey et al., 2020) and experimental (Zhu et al., 2015; Hu et al., 2021) data indicate that destabilization of submembrane MTs in the absence of glucose trigger does not strongly influence the amount of IGs at the periphery. Accordingly, at basal glucose levels, effects of MT destabilization on secretion are not detectable (Zhu et al., 2015; Trogden et al., 2021) unless the accumulated effects over several days are evaluated (Ho et al., 2020). However, destabilization under conditions of active IG retention [glucose-activated IG-plasma membrane association, or docking (Nagamatsu, 2006; Nagamatsu and Ohara-Imaizumi, 2008)], leads to a dramatic IG accumulation at the plasma membrane (Zhu et al., 2015; Hu et al., 2021). We speculate that MT destabilization promotes docking by eliminating fast IG movement and allowing for longer IG dwelling in the proximity of docking molecular machinery (Figure 2B). It is also possible that active transport physically breaks emerging protein interactions and rips some already docked IGs away from the secretion sites. Importantly, IG docking and secretion do not occur randomly across the plasma membrane. Rather, it is allowed only at so-called secretion “hot spots”, cortical/plasma membrane locations where clustered exocytic machinery targets secretion into the bloodstream (Yuan et al., 2015b; Fu et al., 2017; Ohara-Imaizumi et al., 2019). MT-dependent mechanisms restrict functioning of those hot spots: MT destabilization by nocodazole increases the number of actively secreting hot spots per cell (Trogden et al., 2021). In part, the hot spots are activated in otherwise dormant, non-secreting β cell subpopulation. This suggests that the differences in MT stability observed over β cell population in an islet might be one of the mechanisms of functional β-cell heterogeneity, reviewed in (Miranda et al., 2021). MT presence also restricted the number of IGs secreted at each hot-spot location. This implies that if MTs act *via* removal of IGs from the docking sites, they remove all IGs from some secretion loci, and only a percent from others. Exact MT organization and dynamics at secretion hot spots are unknown, and whether it is differential between hot spots, is yet to be understood. It also cannot be excluded that MTs regulate secretion hot-spot machinery through a different, non-IG-transport-dependent mechanism. For example, the turnover of hot-spot structural elements could be regulated by MTs similar to the integrin and membrane receptor recycling (Yoon et al., 2005; Pellinen et al., 2006; Balasubramanian et al., 2007). Along these lines, MTs were shown to promote the localization of clathrin pits to the vicinity of insulin secretion sites, which is necessary for compensatory endocytosis, and, potentially, molecular component turnover during secretion responses (Yuan et al., 2015a).

Considering MT roles in various trafficking processes in a β cell besides IG transport and positioning, it is important to consider functions of MT-dependent transport at earlier stages of insulin biogenesis. MTs are known to promote every stage of protein production and trafficking in the cytoplasm, including mRNA transport, ER shaping, ER-to-Golgi and Golgi-to-ER trafficking, and exit of secretory vesicles from the trans-Golgi network (TGN) (Palmer et al., 2005; Luini et al., 2008; Suter, 2018). It is plausible that all same steps are regulated in β cells and influence insulin production, as suggested by early studies (Malaisse-Lagae et al., 1979). It is indeed true for efficient budding on nascent IGs off the TGN (Figure 2B): under high glucose conditions when IG biogenesis must be intensified, without efficient MT nucleation at the Golgi IG biogenesis fails (Trogden et al., 2019). This means that GDMTs are critical in replenishing IG population after each secretion cycle and maintaining β-cell fitness. This function, likely similar to what was described for post-Golgi carrier formation during constitutive secretion (Polishchuk et al., 2003), indicates an important MT contribution to the positive regulation of insulin secretion at the IG biogenesis stage.

Thus, the MT network in β cells promotes IG availability in the long-term (*via* biogenesis and distribution in the cytoplasm) but restricts IG secretion in the short-term (by withdrawing IGs from secretion sites).

## Microtubule-Dependent Molecular Motors and Their Role in Glucose-Stimulated Insulin Secretion

**FIGURE 3:**
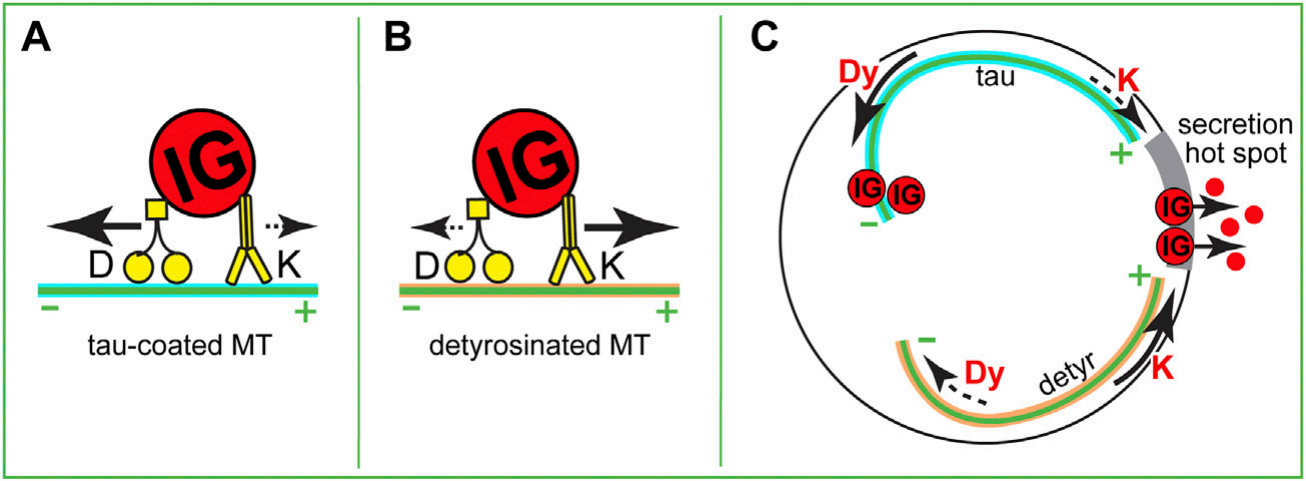
Hypothetical roles of MT-dependent molecular motors in IG transport. **(A,B)** Variants of a tug-of-war between dynein and kinesin-1. **(A)** On a tau-coated MT, dynein overcomes kinesin-1 and transports an IG toward the minus end. **(B)** On a detyrosinated MT, kinesin-1 overcomes dynein and transports an IG toward the plus end. **(C)** A variant of IG delivery-withdrawn regulation. If in high glucose submembrane MTs are partially depolymerized so that the plus ends of remaining MTs are oriented toward a secretion hot spot (gray), different MT modifications could result in either withdrawal (dynein-dependent transport on a tau-coated MT) or delivery (kinesin-1-dependent transport on a detyrosinated MT) of IGs to the secretion sites.

As summarized above, we are starting to understand how MT networks are organized and metabolically tuned in β cells. Besides rearranging the MT geometry, IG transport can be tuned by activation of molecular motors or by changing the capacity of MTs to serve as tracks for specific motors (Figures 3A,B, (Yu et al., 2015; Monroy et al., 2018). MT-dependent motors are recognized by their capacity to move toward the plus- or minus- end of a MT. In a non-differentiated cell with a radial MT network plus-end directed motors drive center-to-periphery (anterograde) transport, while minus-end directed motors drive periphery-to-interior (retrograde) transport. At this point, we do not have a good understanding of MT plus and minus end distributions within complex β-cell MT networks. With largely non-centrosomal long-lived MTs, a significant MT population being parallel to the plasma membrane, and many short MT fragments, it is difficult to predict their polarity. Thus, it is elusive whether plus- or minus-end directed transport will be more efficient in taking IGs to or from the cell border and even less clear, to or from secretion hot spots. To understand specific motor functions, it is important to gather more knowledge on MT polarity and the regulation and function of specific motors in β cells.

The major molecular motor that is thought to drive insulin transport is conventional kinesin, or kinesin-1 (KHC, KIF5b) (Balczon et al., 1992; Varadi et al., 2002; Varadi et al., 2003). It is robustly present at isolated IGs (Balczon et al., 1992), and colocalized with insulin-containing vesicles in β-cell lines (MIN6 & INS-1) (Varadi et al., 2002). Inactivation of kinesin-1 in Min6 cells results in seized IG movement (Varadi et al., 2003). Moreover, a variety of kinesin-1 loss-of-function approaches in cell lines and in mice lead to decreased GSIS levels (Meng et al., 1997; Varadi et al., 2003).

The importance of kinesin-1 for GSIS is consistent with the glucose-dependent regulation of kinesin-1 activity. Interestingly, kinesin-1 heavy chain is heavily phosphorylated at basal (low glucose) conditions (Donelan et al., 2002). Kinesin-1 phosphorylation by a variety of kinases has been associated with inhibited motor activity, data accumulated mostly *in vitro* and in neurons (Morfini et al., 2016). Kinesin-1 becomes dephosphorylated upon high glucose stimulation (Donelan et al., 2002), which is correlated with faster insulin granule movement (McDonald et al., 2009; Zhu et al., 2015).

Collectively, these data strongly indicate that kinesin-1 positively regulated insulin secretion, either *via* promoting the steady-state distribution of IG in the cytoplasm, or by specifically targeting IGs to secretion hot spots. The first option would result from the already described non-directional sub-diffusive transport (Tabei et al., 2013; Zhu et al., 2015). The second option assumes the existence of a MT subset that has an accumulation of MT plus ends at secretion hot spots and favors kinesin-1 movement toward those spots (Figure 3C). Such a subset is yet to be detected in β cells, but it could be created *via* such mechanisms as detyrosination (Figure 3B), MAP7 accumulation, or other MAP/tubulin PTM variations (Barlan et al., 2013; Yu et al., 2015; Monroy et al., 2018).

Very little is known about the role of other MT-dependent motors in insulin transport. Retrograde transport of granules was implicated in the retrieval of granules away from the secretion sites during kiss-and-run exocytosis (Varadi et al., 2003), however, because of the lack of clarity in MT organization and polarity at those sites, it is yet unclear whether it is dynein or another motor that drives such retrieval. It is tempting to extrapolate the role of dynein as a “brake” that slows down plus-end directed movement [tug-of-war mechanism reported for other cell types (Bryantseva and Zhapparova, 2012)] to β cells (Figures 3A,B), however this hypothesis has not been tested yet. Interestingly, dynein activity is dramatically decreased on detyrosinated MTs (McKenney et al., 2016), which would lead to the release of a brake on kinesin-1 movement and make detyrosinated MTs strongly preferred tracks for plus end-directed IG transport (Figure 1B). Another extrapolation from the neuronal scenario calls for testing whether a local accumulation of specific MAPs [e.g. tau enrichment at the peripheral MT bundle: tau regulates the efficiency of several motors, restricting kinesin-1 but not dynein movement (Vershinin et al., 2007; Dixit et al., 2008; Chaudhary et al., 2018; Tan et al., 2019)] defines which motor takes advantage for the movement of IG in a certain direction or to a certain location (Figure 3A). Additionally, a variety of other MT-dependent motors that are expressed in β cells at lower levels might be important for fine-tuning IG transport.

Finally, it is important to consider the role of MTs and MT motors in the transport of other β-cell components, positioning of which can have indirect yet very important effects on insulin secretion. For example, the scaffolding of transcription factor SP1 at MTs by kinesin KIF12 promotes GSIS and glucose homeostasis in mice *via* regulating the oxidative stress (Yang et al., 2014). It is known from other cell models that kinesin-1 can reconfigure the MT network *via* transport and intracellular positioning of MT fragments (Jolly and Gelfand, 2010). The main glucose-processing stations in cells, mitochondria, are positioned at the sites of energy consumption by MT motor-dependent transport [e.g., (Wang and Schwarz, 2009)]. An important process of insulin degradation/turnover could also be dependent on MT transport, since in other cell types, lysosome movement is mediated by both kinesin-1 (Hollenbeck and Swanson, 1990) and dynein motors (Harada et al., 1998; Jordens et al., 2001). MT-motor-dependent transport is also crucial for the organization of many other cellular features which could contribute to β-cell fitness.

## Conclusion

To conclude, we are currently at an exciting nucleation point where increased understanding of MT organization and regulation will inform how GSIS is precisely tuned in endocrine islet β cells. Specifically, we now know that the β-cell MT network is built in a unique configuration. This configuration, surely the MT stability and possibly also the 3D precise arrangement, is varied within the cell population to contribute to β-cell functional heterogeneity. We also know that the MT network is remodeled downstream of glucose in such a way that both MT-dependent insulin biogenesis and secretion are allowed. Yet, mere MT presence serves as a negative regulator, adding to other “filter” mechanisms that prevent insulin over-secretion. Kinesin-1 is specifically activated by glucose to support GSIS, and there is still an intriguing possibility that other MT-dependent motors act in concert with kinesin-1 for the precision and restriction of the response. Thus, future studies to illustrate how MT-regulators and motor proteins interact are essential for a better understanding of β-cell function.

Another intriguing area of future research is dissecting the cooperation and hierarchy of the secretion-restricting “filters”. To this end, depolymerization of the actin cytoskeleton, which strongly promotes GSIS (van Obberghen et al., 1973; Li et al., 1994; Thurmond et al., 2003), also leads to a partial MT network disruption and eliminates MT-dependent regulation of secretion (Zhu et al., 2015). Furthermore, MT depolymerization affects neither glucose-induced calcium influx nor secretion enforced by membrane depolarization (Mourad et al., 2011; Trogden et al., 2021), suggesting that calcium-dependent component of GSIS is not controlled by MTs. At the same time, the intriguing question whether and how calcium-independent mechanisms downstream of glucose are affected by MT presence has received some mixed answers (Mourad et al., 2011; Trogden et al., 2021), indicating a potential for yet-unknown, condition-dependent, cooperation of those pathways. Studies on actin-MT cross-regulation in IG localization and Ca^2+^-dependent vesicle-plasma membrane fusion should not only help with our understanding of diabetes, but also serve as a prototype in understanding how different cell types leverage the regulation and configuration of MTs to serve distinct physiological functions.

## Abbreviations

FIB-SEM, focused ion beam scanning electron microscopy; GDMT, Golgi-derived MT; GSIS, glucose-stimulated insulin secretion; IG, insulin granule; MT, microtubule; MAP, MT-associated protein.

## References

[B1] Alejandro E. U. Gregg B. Blandino-Rosano M. Cras-Méneur C. Bernal-Mizrachi E. (2015). Natural History of β-cell Adaptation and Failure in Type 2 Diabetes. Mol. Aspects Med. 42, 19–41. 10.1016/j.mam.2014.12.002 25542976 PMC4404183

[B2] Arous C. Halban P. A. (2015). The Skeleton in the Closet: Actin Cytoskeletal Remodeling in β-cell Function. Am. J. Physiology-Endocrinology Metabolism 309 (7), E611–E620. 10.1152/ajpendo.00268.2015 26286869

[B3] Avrahami D. Klochendler A. Dor Y. Glaser B. (2017). Beta Cell Heterogeneity: an Evolving Concept. Diabetologia 60 (8), 1363–1369. 10.1007/s00125-017-4326-z 28597073 PMC5554543

[B4] Balasubramanian N. Scott D. W. Castle J. D. Casanova J. E. Schwartz M. A. (2007). Arf6 and Microtubules in Adhesion-dependent Trafficking of Lipid Rafts. Nat. Cell Biol. 9 (12), 1381–1391. 10.1038/ncb1657 18026091 PMC2715295

[B5] Balczon R. Overstreet K. A. Zinkowski R. P. Haynes A. Appel M. (1992). The Identification, Purification, and Characterization of a Pancreatic Beta-Cell Form of the Microtubule Adenosine Triphosphatase Kinesin. Endocrinology 131 (1), 331–336. 10.1210/endo.131.1.1612013 1612013

[B6] Barbier P. Zejneli O. Martinho M. Lasorsa A. Belle V. Smet-Nocca C. (2019). Role of Tau as a Microtubule-Associated Protein: Structural and Functional Aspects. Front. Aging Neurosci. 11, 204. 10.3389/fnagi.2019.00204 31447664 PMC6692637

[B7] Barlan K. Lu W. Gelfand V. I. (2013). The Microtubule-Binding Protein Ensconsin Is an Essential Cofactor of Kinesin-1. Curr. Biol. 23 (4), 317–322. 10.1016/j.cub.2013.01.008 23394833 PMC3580027

[B8] Benninger R. K. P. Kravets V. (2022). The Physiological Role of β-cell Heterogeneity in Pancreatic Islet Function. Nat. Rev. Endocrinol. 18 (1), 9–22. 10.1038/s41574-021-00568-0 34667280 PMC8915749

[B9] Bicek A. D. Tüzel E. Demtchouk A. Uppalapati M. Hancock W. O. Kroll D. M. (2009). Anterograde Microtubule Transport Drives Microtubule Bending in LLC-PK1 Epithelial Cells. MBoC 20 (12), 2943–2953. 10.1091/mbc.E08-09-0909 19403700 PMC2695801

[B10] Boyd A. E. 3rd Bolton W. E. Brinkley B. R. (1982). Microtubules and Beta Cell Function: Effect of Colchicine on Microtubules and Insulin Secretion *In Vitro* by Mouse Beta Cells. J. Cell Biol. 92 (2), 425–434. 10.1083/jcb.92.2.425 7037795 PMC2112066

[B11] Bracey K. M. Ho K.-H. Yampolsky D. Gu G. Kaverina I. Holmes W. R. (2020). Microtubules Regulate Localization and Availability of Insulin Granules in Pancreatic Beta Cells. Biophysical J. 118 (1), 193–206. 10.1016/j.bpj.2019.10.031 PMC6950633 31839261

[B12] Bryantseva S. A. Zhapparova O. N. (2012). Bidirectional Transport of Organelles: Unity and Struggle of Opposing Motors. Cell. Biol. Int. 36 (1), 1–6. 10.1042/CBI20110413 22142363

[B13] Byers H. R. Fujiwara K. Porter K. R. (1980). Visualization of Microtubules of Cells *In Situ* by Indirect Immunofluorescence. Proc. Natl. Acad. Sci. U.S.A. 77 (11), 6657–6661. 10.1073/pnas.77.11.6657 6935678 PMC350346

[B14] Chatterjee Bhowmick D. Ahn M. Oh E. Veluthakal R. Thurmond D. C. (2021). Conventional and Unconventional Mechanisms by Which Exocytosis Proteins Oversee β-cell Function and Protection. Ijms 22 (4), 1833. 10.3390/ijms22041833 33673206 PMC7918544

[B15] Chaudhary A. R. Berger F. Berger C. L. Hendricks A. G. (2018). Tau Directs Intracellular Trafficking by Regulating the Forces Exerted by Kinesin and Dynein Teams. Traffic 19 (2), 111–121. 10.1111/tra.12537 29077261 PMC5783771

[B16] Dean P. M. (1973). Ultrastructural Morphometry of the Pancreatic ?-cell. Diabetologia 9 (2), 115–119. 10.1007/BF01230690 4577291

[B17] Dixit R. Ross J. L. Goldman Y. E. Holzbaur E. L. F. (2008). Differential Regulation of Dynein and Kinesin Motor Proteins by Tau. Science 319 (5866), 1086–1089. 10.1126/science.1152993 18202255 PMC2866193

[B18] Donelan M. J. Morfini G. Julyan R. Sommers S. Hays L. Kajio H. (2002). Ca2+-dependent Dephosphorylation of Kinesin Heavy Chain on β-Granules in Pancreatic β-Cells. J. Biol. Chem. 277 (27), 24232–24242. 10.1074/jbc.m203345200 11978799

[B19] Fu J. Dai X. Plummer G. Suzuki K. Bautista A. Githaka J. M. (2017). Kv2.1 Clustering Contributes to Insulin Exocytosis and Rescues Human β-Cell Dysfunction. Diabetes 66 (7), 1890–1900. 10.2337/db16-1170 28607108 PMC5482075

[B20] Fu Z. R. Gilbert E. Liu D. (2013). Regulation of Insulin Synthesis and Secretion and Pancreatic Beta-Cell Dysfunction in Diabetes. Curr. Diabetes Rev. 9 (1), 25–53. 10.2174/157339913804143225 22974359 PMC3934755

[B21] Gandasi N. R. Yin P. Omar-Hmeadi M. Ottosson Laakso E. Vikman P. Barg S. (2018). Glucose-Dependent Granule Docking Limits Insulin Secretion and Is Decreased in Human Type 2 Diabetes. Cell Metab. 27 (2), 470–478. 10.1016/j.cmet.2017.12.017 29414688

[B22] Gao N. White P. Doliba N. Golson M. L. Matschinsky F. M. Kaestner K. H. (2007). Foxa2 Controls Vesicle Docking and Insulin Secretion in Mature β Cells. Cell Metab. 6 (4), 267–279. 10.1016/j.cmet.2007.08.015 17908556

[B23] Gundersen G. G. Khawaja S. Bulinski J. C. (1987). Postpolymerization Detyrosination of Alpha-Tubulin: a Mechanism for Subcellular Differentiation of Microtubules. J. Cell Biol. 105 (1), 251–264. 10.1083/jcb.105.1.251 2886509 PMC2114889

[B24] Harada A. Takei Y. Kanai Y. Tanaka Y. Nonaka S. Hirokawa N. (1998). Golgi Vesiculation and Lysosome Dispersion in Cells Lacking Cytoplasmic Dynein. J. Cell Biol. 141 (1), 51–59. 10.1083/jcb.141.1.51 9531547 PMC2132725

[B25] Heaslip A. T. Nelson S. R. Lombardo A. T. Beck Previs S. Armstrong J. Warshaw D. M. (2014). Cytoskeletal Dependence of Insulin Granule Movement Dynamics in INS-1 Beta-Cells in Response to Glucose. PLoS One 9 (10), e109082. 10.1371/journal.pone.0109082 25310693 PMC4195697

[B26] Ho K.-H. Yang X. Osipovich A. B. Cabrera O. Hayashi M. L. Magnuson M. A. (2020). Glucose Regulates Microtubule Disassembly and the Dose of Insulin Secretion via Tau Phosphorylation. Diabetes 69 (9), 1936–1947. 10.2337/db19-1186 32540877 PMC7458041

[B27] Hoboth P. Müller A. Ivanova A. Mziaut H. Dehghany J. Sönmez A. (2015). Aged Insulin Granules Display Reduced Microtubule-dependent Mobility and Are Disposed within Actin-Positive Multigranular Bodies. Proc. Natl. Acad. Sci. U.S.A. 112 (7), E667–E676. 10.1073/pnas.1409542112 25646459 PMC4343180

[B28] Hollenbeck P. J. Swanson J. A. (1990). Radial Extension of Macrophage Tubular Lysosomes Supported by Kinesin. Nature 346 (6287), 864–866. 10.1038/346864a0 1697403

[B29] Hou J. C. Min L. Pessin J. E. (2009). Chapter 16 Insulin Granule Biogenesis, Trafficking and Exocytosis. Vitam. Horm. 80, 473–506. 10.1016/S0083-6729(08)00616-X 19251047 PMC4324607

[B30] Howell S. L. Hii C. S. Shaikh S. Tyhurst M. (1982). Effects of Taxol and Nocodazole on Insulin Secretion from Isolated Rat Islets of Langerhans. Biosci. Rep. 2 (10), 795–801. 10.1007/BF01114939 6129005

[B31] Hu R. Zhu X. Yuan M. Ho K.-H. Kaverina I. Gu G. (2021). Microtubules and Gαo-Signaling Modulate the Preferential Secretion of Young Insulin Secretory Granules in Islet β Cells via Independent Pathways. PLoS One 16 (7), e0241939. 10.1371/journal.pone.0241939 34292976 PMC8297875

[B32] Hudish L. I. Reusch J. E. B. Sussel L. (2019). β Cell Dysfunction during Progression of Metabolic Syndrome to Type 2 Diabetes. J. Clin. Invest. 129 (10), 4001–4008. 10.1172/JCI129188 31424428 PMC6763241

[B33] Idevall-Hagren O. Tengholm A. (2020). Metabolic Regulation of Calcium Signaling in Beta Cells. Seminars Cell & Dev. Biol. 103, 20–30. 10.1016/j.semcdb.2020.01.008 32085965

[B34] Jolly A. L. Gelfand V. I. (2010). Cytoplasmic Microtubule Sliding. Commun. Integr. Biol. 3 (6), 589–591. 10.4161/cib.3.6.13212 21331248 PMC3038072

[B35] Jordens I. Fernandez-Borja M. Marsman M. Dusseljee S. Janssen L. Calafat J. (2001). The Rab7 Effector Protein RILP Controls Lysosomal Transport by Inducing the Recruitment of Dynein-Dynactin Motors. Curr. Biol. 11 (21), 1680–1685. 10.1016/s0960-9822(01)00531-0 11696325

[B36] Kalwat M. A. Thurmond D. C. (2013). Signaling Mechanisms of Glucose-Induced F-Actin Remodeling in Pancreatic Islet β Cells. Exp. Mol. Med. 45, e37. 10.1038/emm.2013.73 23969997 PMC3789261

[B37] Kasai K. Fujita T. Gomi H. Izumi T. (2008). Docking Is Not a Prerequisite but a Temporal Constraint for Fusion of Secretory Granules. Traffic 9, 1191–1203. 10.1111/j.1600-0854.2008.00744.x 18397364

[B38] Khawaja S. Gundersen G. G. Bulinski J. C. (1988). Enhanced Stability of Microtubules Enriched in Detyrosinated Tubulin Is Not a Direct Function of Detyrosination Level. J. Cell Biol. 106 (1), 141–149. 10.1083/jcb.106.1.141 3276710 PMC2114950

[B39] Lacy P. E. Walker M. M. Joan Fink C. (1972). Perifusion of Isolated Rat Islets *In Vitro*: Participation of the Microtubular System in the Biphasic Release of Insulin. Diabetes 21 (10), 987–998. 10.2337/diab.21.10.987 4561331

[B40] Li G. Rungger-Brändle E. Just I. Jonas J. C. Aktories K. Wollheim C. B. (1994). Effect of Disruption of Actin Filaments by Clostridium Botulinum C2 Toxin on Insulin Secretion in HIT-T15 Cells and Pancreatic Islets. MBoC 5 (11), 1199–1213. 10.1091/mbc.5.11.1199 7865885 PMC301146

[B41] Lindwall G. Cole R. D. (1984). Phosphorylation Affects the Ability of Tau Protein to Promote Microtubule Assembly. J. Biol. Chem. 259 (8), 5301–5305. 10.1016/s0021-9258(17)42989-9 6425287

[B42] Luini A. Mironov A. A. Polishchuk E. V. Polishchuk R. S. (2008). Morphogenesis of Post-golgi Transport Carriers. Histochem Cell Biol. 129 (2), 153–161. 10.1007/s00418-007-0365-8 18214517 PMC2228382

[B43] Malaisse-Lagae F. Amherdt M. Ravazzola M. Sener A. Hutton J. C. Orci L. (1979). Role of Microtubules in the Synthesis, Conversion, and Release of (Pro)insulin. A Biochemical and Radioautographic Study in Rat Islets. J. Clin. Invest.. 63 (6), 1284–1296. 10.1172/JCI109423 376557 PMC372077

[B44] McDonald A. Fogarty S. Leclerc I. Hill E. V. Hardie D. G. Rutter G. A. (2009). Control of Insulin Granule Dynamics by AMPK Dependent KLC1 Phosphorylation. Islets 1 (3), 198–209. 10.4161/isl.1.3.9608 21099273

[B45] McKenney R. J. Huynh W. Vale R. D. Sirajuddin M. (2016). Tyrosination of α‐tubulin Controls the Initiation of Processive Dynein-Dynactin Motility. EMBO J. 35 (11), 1175–1185. 10.15252/embj.201593071 26968983 PMC4888239

[B46] Meng Y. X. Wilson G. W. Avery M. C. Varden C. H. Balczon R. (1997). Suppression of the Expression of a Pancreatic β-Cell Form of the Kinesin Heavy Chain by Antisense Oligonucleotides Inhibits Insulin Secretion from Primary Cultures of Mouse β-Cells*. Endocrinology 138 (5), 1979–1987. 10.1210/endo.138.5.5139 9112396

[B47] Miranda M. A. Macias-Velasco J. F. Lawson H. A. (2021). Pancreatic β-cell Heterogeneity in Health and Diabetes: Classes, Sources, and Subtypes. Am. J. Physiology-Endocrinology Metabolism 320 (4), E716–E731. 10.1152/ajpendo.00649.2020 PMC8238131 33586491

[B48] Monroy B. Y. Sawyer D. L. Ackermann B. E. Borden M. M. Tan T. C. Ori-McKenney K. M. (2018). Competition between Microtubule-Associated Proteins Directs Motor Transport. Nat. Commun. 9 (1), 1487. 10.1038/s41467-018-03909-2 29662074 PMC5902456

[B49] Morfini G. Schmidt N. Weissmann C. Pigino G. Kins S. (2016). Conventional Kinesin: Biochemical Heterogeneity and Functional Implications in Health and Disease. Brain Res. Bull. 126 (Pt 3), 347–353. 10.1016/j.brainresbull.2016.06.009 27339812

[B50] Mourad N. I. Nenquin M. Henquin J.-C. (2011). Metabolic Amplification of Insulin Secretion by Glucose Is Independent of β-cell Microtubules. Am. J. Physiology-Cell Physiology 300 (3), C697–C706. 10.1152/ajpcell.00329.2010 21178111

[B51] Müller A. Schmidt D. Xu C. S. Pang S. D’Costa J. V. Kretschmar S. (2021). 3D FIB-SEM Reconstruction of Microtubule-Organelle Interaction in Whole Primary Mouse β Cells. J. Cell Biol. 220 (2), e202010039. 10.1083/jcb.202010039 33326005 PMC7748794

[B52] Nagamatsu S. Ohara-Imaizumi M. (2008). Imaging Exocytosis of Single Insulin Secretory Granules with TIRF Microscopy. Methods Mol. Biol. 440, 259–268. 10.1007/978-1-59745-178-9_20 18369952

[B53] Nagamatsu S. (2006). TIRF Microscopy Analysis of the Mechanism of Insulin Exocytosis. Endocr. J. 53 (4), 433–440. 10.1507/endocrj.kr-75 16807501

[B54] Nasteska D. Hodson D. J. (2018). The Role of Beta Cell Heterogeneity in Islet Function and Insulin Release. J. Mol. Endocrinol. 61 (1), R43–R60. 10.1530/JME-18-0011 29661799 PMC5976077

[B55] Nessa A. Rahman S. A. Hussain K. (2016). Hyperinsulinemic Hypoglycemia - the Molecular Mechanisms. Front. Endocrinol. 7, 29. 10.3389/fendo.2016.00029 PMC4815176 27065949

[B56] Odde D. J. Ma L. Briggs A. H. DeMarco A. Kirschner M. W. (1999). Microtubule Bending and Breaking in Living Fibroblast Cells. J. Cell Sci. 112 (Pt 19), 3283–3288. 10.1242/jcs.112.19.3283 10504333

[B57] Ohara-Imaizumi M. Aoyagi K. Ohtsuka T. (2019). Role of the Active Zone Protein, ELKS, in Insulin Secretion from Pancreatic β-cells. Mol. Metab. 27, S81–S91. 10.1016/j.molmet.2019.06.017 PMC6768504 31500835

[B58] Olofsson C. S. Göpel S. O. Barg S. Galvanovskis J. Ma X. Salehi A. (2002). Fast Insulin Secretion Reflects Exocytosis of Docked Granules in Mouse Pancreatic B-Cells. Pflügers Arch. - Eur. J. Physiol. 444 (1-2), 43–51. 10.1007/s00424-002-0781-5 11976915

[B59] Omar-Hmeadi M. Idevall-Hagren O. (2021). Insulin Granule Biogenesis and Exocytosis. Cell. Mol. Life Sci. 78 (5), 1957–1970. 10.1007/s00018-020-03688-4 33146746 PMC7966131

[B60] Palmer K. J. Watson P. Stephens D. J. (2005). The Role of Microtubules in Transport between the Endoplasmic Reticulum and Golgi Apparatus in Mammalian Cells. Biochem. Soc. Symp. 72 (72), 1–13. 10.1042/bss0720001 15649125

[B61] Pellinen T. Arjonen A. Vuoriluoto K. Kallio K. Fransen J. A. M. Ivaska J. (2006). Small GTPase Rab21 Regulates Cell Adhesion and Controls Endosomal Traffic of β1-integrins. J. Cell Biol. 173 (5), 767–780. 10.1083/jcb.200509019 16754960 PMC2063892

[B62] Polishchuk E. V. Di Pentima A. Luini A. Polishchuk R. S. (2003). Mechanism of Constitutive Export from the Golgi: Bulk Flow via the Formation, Protrusion, and *En Bloc* Cleavage of Largetrans-Golgi Network Tubular Domains. MBoC 14 (11), 4470–4485. 10.1091/mbc.e03-01-0033 12937271 PMC266766

[B63] Portran D. Schaedel L. Xu Z. Théry M. Nachury M. V. (2017). Tubulin Acetylation Protects Long-Lived Microtubules against Mechanical Ageing. Nat. Cell Biol. 19 (4), 391–398. 10.1038/ncb3481 28250419 PMC5376231

[B64] Ramos L. S. Zippin J. H. Kamenetsky M. Buck J. Levin L. R. (2008). Glucose and GLP-1 Stimulate cAMP Production via Distinct Adenylyl Cyclases in INS-1E Insulinoma Cells. J. Gen. Physiol. 132 (3), 329–338. 10.1085/jgp.200810044 18695009 PMC2518727

[B65] Renström E. Eliasson L. Rorsman P. (1997). Protein Kinase A-dependent and -independent Stimulation of Exocytosis by cAMP in Mouse Pancreatic B-Cells. J. Physiol. 502 (Pt 1), 105–118. 10.1111/j.1469-7793.1997.105bl.x 9234200 PMC1159575

[B66] Roll-Mecak A. Vale R. D. (2006). Making More Microtubules by Severing: a Common Theme of Noncentrosomal Microtubule Arrays? J. Cell Biol. 175 (6), 849–851. 10.1083/jcb.200611149 17178905 PMC2064694

[B67] Rorsman P. Renstrom E. (2003). Insulin Granule Dynamics in Pancreatic Beta Cells. Diabetologia 46 (8), 1029–1045. 10.1007/s00125-003-1153-1 12879249

[B68] Sanders A. A. W. M. Kaverina I. (2015). Nucleation and Dynamics of Golgi-Derived Microtubules. Front. Neurosci. 9, 431. 10.3389/fnins.2015.00431 26617483 PMC4639703

[B69] Straube A. Hause G. Fink G. Steinberg G. (2006). Conventional Kinesin Mediates Microtubule-Microtubule Interactions *In Vivo* . MBoC 17 (2), 907–916. 10.1091/mbc.e05-06-0542 16339079 PMC1356599

[B70] Suter B. (2018). RNA Localization and Transport. Biochimica Biophysica Acta (BBA) - Gene Regul. Mech. 1861 (10), 938–951. 10.1016/j.bbagrm.2018.08.004 30496039

[B71] Tabei S. M. A. Burov S. Kim H. Y. Kuznetsov A. Huynh T. Jureller J. (2013). Intracellular Transport of Insulin Granules Is a Subordinated Random Walk. Proc. Natl. Acad. Sci. U.S.A. 110 (13), 4911–4916. 10.1073/pnas.1221962110 23479621 PMC3612641

[B72] Tan R. Lam A. J. Tan T. Han J. Nowakowski D. W. Vershinin M. (2019). Microtubules Gate Tau Condensation to Spatially Regulate Microtubule Functions. Nat. Cell Biol. 21 (9), 1078–1085. 10.1038/s41556-019-0375-5 31481790 PMC6748660

[B73] Thurmond D. C. Gonelle-Gispert C. Furukawa M. Halban P. A. Pessin J. E. (2003). Glucose-Stimulated Insulin Secretion Is Coupled to the Interaction of Actin with the T-SNARE (Target Membrane SolubleN-Ethylmaleimide-Sensitive Factor Attachment Protein Receptor Protein) Complex. Mol. Endocrinol. 17 (4), 732–742. 10.1210/me.2002-0333 12554769

[B74] Trogden K. P. Lee J. Bracey K. M. Ho K.-H. McKinney H. Zhu X. (2021). Microtubules Regulate Pancreatic β-cell Heterogeneity via Spatiotemporal Control of Insulin Secretion Hot Spots. Elife 10. 10.7554/eLife.59912 PMC8635970 34783306

[B75] Trogden K. P. Zhu X. Lee J. S. Wright C. V. E. Gu G. Kaverina I. (2019). Regulation of Glucose-Dependent Golgi-Derived Microtubules by cAMP/EPAC2 Promotes Secretory Vesicle Biogenesis in Pancreatic β Cells. Curr. Biol. 29 (14), 2339–2350 e5. 10.1016/j.cub.2019.06.032 31303487 PMC6698911

[B76] van Obberghen E. Somers G. Devis G. Vaughan G. D. Malaisse-Lagae F. Orci L. (1973). Dynamics of Insulin Release and Microtubular-Microfilamentous System. I. Effect of Cytochalasin B. J. Clin. Invest.. 52 (5), 1041–1051. 10.1172/JCI107269 4573352 PMC302358

[B77] Varadi A. Ainscow E. K. Allan V. J. Rutter G. A. (2002). Involvement of Conventional Kinesin in Glucose-Stimulated Secretory Granule Movements and Exocytosis in Clonal Pancreatic Beta-Cells. J. Cell Sci. 115 (Pt 21), 4177–4189. 10.1242/jcs.00083 12356920

[B78] Varadi A. Tsuboi T. Johnson-Cadwell L. I. Allan V. J. Rutter G. A. (2003). Kinesin I and Cytoplasmic Dynein Orchestrate Glucose-Stimulated Insulin-Containing Vesicle Movements in Clonal MIN6 β-cells. Biochem. Biophysical Res. Commun. 311 (2), 272–282. S0006291X03020473 [pii]. 10.1016/j.bbrc.2003.09.208 14592410

[B79] Varadi A. Tsuboi T. Rutter G. A. (2005). Myosin Va Transports Dense Core Secretory Vesicles in Pancreatic MIN6 β-Cells. MBoC 16 (6), 2670–2680. 10.1091/mbc.e04-11-1001 15788565 PMC1142415

[B80] Veluthakal R. Thurmond D. C. (2021). Emerging Roles of Small GTPases in Islet β-Cell Function. Cells 10 (6), 1503. 10.3390/cells10061503 34203728 PMC8232272

[B81] Verhage M. Sørensen J. B. (2008). Vesicle Docking in Regulated Exocytosis. Traffic 9 (9), 1414–1424. 10.1111/j.1600-0854.2008.00759.x 18445120

[B82] Vershinin M. Carter B. C. Razafsky D. S. King S. J. Gross S. P. (2007). Multiple-motor Based Transport and its Regulation by Tau. Proc. Natl. Acad. Sci. U.S.A. 104 (1), 87–92. 10.1073/pnas.0607919104 17190808 PMC1765483

[B83] Wang X. Schwarz T. L. (2009). The Mechanism of Ca2+-Dependent Regulation of Kinesin-Mediated Mitochondrial Motility. Cell 136 (1), 163–174. 10.1016/j.cell.2008.11.046 19135897 PMC2768392

[B84] Wang Z. Thurmond D. C. (2009). Mechanisms of Biphasic Insulin-Granule Exocytosis - Roles of the Cytoskeleton, Small GTPases and SNARE Proteins. J. Cell Sci. 122 (Pt 7), 893–903. 10.1242/jcs.034355 19295123 PMC2720925

[B85] Yang W. Tanaka Y. Bundo M. Hirokawa N. (2014). Antioxidant Signaling Involving the Microtubule Motor KIF12 Is an Intracellular Target of Nutrition Excess in Beta Cells. Dev. Cell 31 (2), 202–214. 10.1016/j.devcel.2014.08.028 25373778

[B86] Yoon S.-O. Shin S. Mercurio A. M. (2005). Hypoxia Stimulates Carcinoma Invasion by Stabilizing Microtubules and Promoting the Rab11 Trafficking of the α6β4 Integrin. Cancer Res. 65 (7), 2761–2769. 10.1158/0008-5472.CAN-04-4122 15805276

[B87] Yu I. Garnham C. P. Roll-Mecak A. (2015). Writing and Reading the Tubulin Code. J. Biol. Chem. 290 (28), 17163–17172. 10.1074/jbc.R115.637447 25957412 PMC4498056

[B88] Yuan T. Liu L. Zhang Y. Wei L. Zhao S. Zheng X. (2015a). Diacylglycerol Guides the Hopping of Clathrin-Coated Pits along Microtubules for Exo-Endocytosis Coupling. Dev. Cell 35 (1), 120–130. 10.1016/j.devcel.2015.09.004 26439397

[B89] Yuan T. Lu J. Zhang J. Zhang Y. Chen L. (2015b). Spatiotemporal Detection and Analysis of Exocytosis Reveal Fusion "hotspots" Organized by the Cytoskeleton in Endocrine Cells. Biophysical J. 108 (2), 251–260. 10.1016/j.bpj.2014.11.3462 PMC4302205 25606674

[B90] Zhao A. Ohara-Imaizumi M. Brissova M. Benninger R. K. P. Xu Y. Hao Y. (2010). Gαo Represses Insulin Secretion by Reducing Vesicular Docking in Pancreatic β-Cells. Diabetes 59 (10), 2522–2529. 10.2337/db09-1719 20622165 PMC3279551

[B91] Zhu X. Hu R. Brissova M. Stein R. W. Powers A. C. Gu G. (2015). Microtubules Negatively Regulate Insulin Secretion in Pancreatic β Cells. Dev. Cell 34 (6), 656–668. 10.1016/j.devcel.2015.08.020 26418295 PMC4594944

[B92] Zhu X. Kaverina I. (2013). Golgi as an MTOC: Making Microtubules for its Own Good. Histochem Cell Biol. 140 (3), 361–367. 10.1007/s00418-013-1119-4 23821162 PMC3748218

